# Novel drug candidates for blast phase chronic myeloid leukemia from high-throughput drug sensitivity and resistance testing

**DOI:** 10.1038/bcj.2015.30

**Published:** 2015-05-01

**Authors:** P O Pietarinen, T Pemovska, M Kontro, B Yadav, J P Mpindi, E I Andersson, M M Majumder, H Kuusanmäki, P Koskenvesa, O Kallioniemi, K Wennerberg, C A Heckman, S Mustjoki, K Porkka

**Affiliations:** 1 Hematology Research Unit Helsinki, University of Helsinki and Department of Hematology, Comprehensive Cancer Center, Helsinki University Hospital, Helsinki, Finland; 2 Institute for Molecular Medicine Finland, FIMM, University of Helsinki, Helsinki, Finland

## Abstract

Chronic myeloid leukemia in blast crisis (CML BC) remains a challenging disease to treat despite the introduction and advances in tyrosine kinase inhibitor (TKI) therapy. In this study we set out to identify novel candidate drugs for CML BC by using an unbiased high-throughput drug testing platform. We used three CML cell lines representing different types of CML blast phases (K562, EM-2 and MOLM-1) and primary leukemic cells from three CML BC patients. Profiling of drug responses was performed with a drug sensitivity and resistance testing platform comprising 295 anticancer agents. Overall, drug sensitivity scores and the drug response profiles of cell line and primary cell samples correlated well and were distinct from other types of leukemia samples. The cell lines were highly sensitive to TKIs and the clinically TKI-resistant patient samples were also resistant *ex vivo*. Comparison of cell line and patient sample data identified new candidate drugs for CML BC, such as vascular endothelial growth factor receptor and nicotinamide phosphoribosyltransferase inhibitors. Our results indicate that these drugs in particular warrant further evaluation by analyzing a larger set of primary patient samples. The results also pave way for designing rational combination therapies.

## Introduction

Tyrosine kinase inhibitor (TKI) therapy in chronic myeloid leukemia (CML) is arguably the best example of successful targeted cancer treatment. The discovery of the Philadelphia chromosome and the role of BCR-ABL1 kinase in CML pathogenesis led to the development of the first approved TKI imatinib, which has remarkably improved the prognosis and survival of CML patients.^1^ Imatinib is widely used as a first-line therapy together with second-generation TKIs dasatinib and nilotinib, which are also applicable as second-line therapy for patients who become resistant or intolerant to imatinib.^2, 3^

Despite the treatment breakthroughs in chronic phase CML, advanced phase and blast crisis (BC) remain a therapeutic challenge.^4^ Owing to the more aggressive nature of advanced phase CML, patients tend to respond less favorably to TKI treatment. When first-line TKI treatment fails the options available are few, from higher dosages of TKI to allogeneic hematopoietic stem cell transplantation. None of the current treatment options are significantly effective in CML BC. Hence, there is a clear need for identification of novel drug therapies for CML BC patients.

Sequencing of cancer genomes has become a popular choice in characterizing individual cancer patients in the search for new druggable targets. However, genetic findings do not always translate to efficacious drug therapy. Our recent study in acute myeloid leukemia (AML)^5^ presents a new concept of individualized systems medicine, which emphasizes high-throughput drug sensitivity and resistance testing (DSRT) of primary patient samples to profile patient cancer cells and identify personalized treatment strategies. With this novel approach we characterized drug responses in CML BC cell lines and primary patient samples, and aimed to identify new potential candidates for the treatment of CML BC. Our results indicate that both primary CML BC and cell line samples display a unique drug sensitivity pattern, which differs from other leukemia types. Furthermore, novel candidate drugs for CML BC such as vascular endothelial growth factor receptor (VEGFR) and nicotinamide phosphoribosyltransferase (NAMPT) inhibitors were discovered from the DSRT analysis.

## Materials and methods

### Study patients

The bone marrow aspirates or peripheral blood samples (leukemic cells) and skin biopsies (noncancerous cells for germline genomic information) from three CML patients and nine different healthy donors (controls) were obtained after informed consent with approval from local Institutional Review Boards (No. 239/13/03/00/2010, 303/13/03/01/2011) and in accordance with the Declaration of Helsinki. Patient characteristics are summarized in Table 1.

### Cell lines

Human CML BC cell lines (K562, EM-2 and MOLM-1) were obtained from Deutsche Sammlung von Mikroorganismen und Zellkulturen Gmbh (http://www.dsmz.de/). K562 cell line has erythroleukemic (resembles both undifferentiated granulocytes and erythrocytes), MOLM-1 cell line megakaryocytic and EM-2 myeloid features.^6, 7, 8^ Cells were maintained according to the manufacturer's instructions at 37 °C and 5% CO_2_ in RPMI 1640 supplemented with 10% (K562 and EM-2) or 20% (MOLM-1) fetal bovine serum, L-glutamine and penicillin–streptomycin. Collection of drug sensitivity score (DSS) values from >150 cell lines served as a control group for CML BC cell line results (the collection includes AML, breast cancer, ovarian cancer, head and neck cancer, pancreatic cancer and prostate cancer cell lines).

### Primary patient cells

Mononuclear cells were separated from bone marrow aspirates or peripheral blood of three patients and nine healthy donors using Ficoll density gradient separation (FicollPaque PREMIUM; GE Healthcare, Pittsburgh, PA, USA). Mononuclear cells were counted using a hemocytometer and suspended in mononuclear cell medium (PromoCell, Heidelberg, Germany) supplemented with 0.5 μg/ml gentamicin and 2.5 μg/ml amphotericin B before DSRT. Blast counts in tested patient samples 1, 2 and 3 were 40%, 86% and 75%, respectively.

### Drug sensitivity and resistance testing

DSRT protocol has been described previously.^5^ Mononuclear cell medium was used with patient primary cells and healthy controls. Cell line assays were done with the corresponding cell culture medium.

The drug collection used in these studies contained 295 different substances and covered the majority of US Food and Drug Administration/European Medicines Agency-approved anticancer drugs, as well as emerging investigational and preclinical compounds covering a wide range of molecular targets (Supplementary Table 1). The compounds were obtained from the National Cancer Institute Drug Testing Program and commercial chemical vendors. Briefly, the drugs were preplated in a 384-well plate in five different concentrations in a 10 000-fold concentration range and primary cells added at 10 000 cells per well or cell lines at a predetermined number to ensure that each was in growth phase at the end of the assay. All plates were incubated in a humidified environment at 37 °C and 5% CO_2_ for 72 h. Cell viability was measured using CellTiter-Glo luminescent assay (Promega, Madison, WI, USA) according to the manufacturer's instructions with a PHERAstar FS (BMG Labtech, Ortenberg, Germany) or Paradigm (Molecular Devices, Sunnyvale, CA, USA) plate reader. Dose–response curves were generated on the basis of the viability readouts.

### Drug sensitivity analysis

DSS is an integrative and robust drug response model based on normalized area under the curve by taking all four curve fitting parameters into account.^5, 9^ DSS values were further normalized against the median values from healthy controls or from cell line reference data for patient and cell line samples, respectively, to obtain selective DSS, which was then used to measure leukemia-specific drug sensitivity. Drugs with selective DSS values >5 were considered selective and >10 highly selective to tested cells.

In addition to CML BC samples, we used the DSS of different types of leukemia samples in clustering analysis (AML *n*=10, Ph+ ALL *n*=3, T-ALL *n*=3 and B-ALL *n*=3).

### Sequencing

All patient primary cell samples were Sanger sequenced for *BCR-ABL1* mutations after diagnosis. Identified mutations were confirmed by exome sequencing for patient samples 1 and 2 at the time of DSRT sampling.

### Comparison with CGP and CCLE data

The drug response data from Cancer Genome Project (CGP)^10^ and Cancer Cell Line Encyclopedia (CCLE)^11^ studies were further analyzed. DSS was calculated for each drug response profile to compare data from our study with CGP and CCLE data based on the standardized drug response metrics used in our study. The nonparametric Spearman's rank correlation coefficient was used to evaluate the similarity of drug response profiles across the three data sets.

### Statistical analysis

The nonparametric Spearman's rank correlation coefficient was calculated with SPSS Statistics software (version 22, IBM, Armonk, NY, USA). *P*-values for all cell line and patient primary cell sample correlation pairs were <0.0001. Clustering of the drug sensitivity profiles across the cell line and patient samples were performed using unsupervised hierarchical complete-linkage clustering using Spearman and Euclidean distance measures of the drug and sample profiles, respectively. All correlation and clustering analyses were performed using DSS profiles of the cell line and primary patient cell samples.

## Results

### BCR-ABL1 and VEGFR inhibitors highly selective in CML BC cell lines

**Figure 1:**
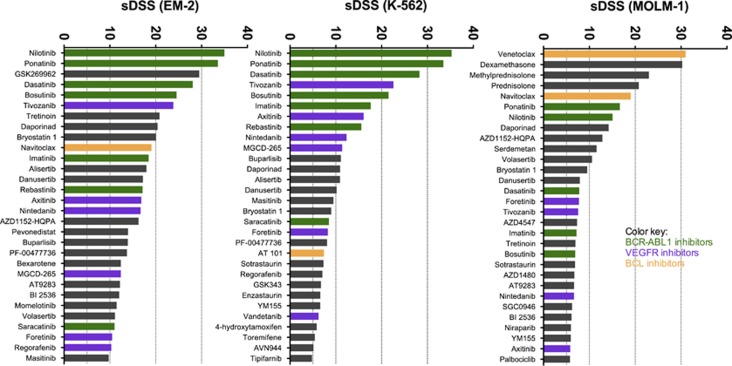
Selective DSS (sDSS) of cell lines (top 30) in descending order. Classic cytostatic and cytotoxic drugs were filtered out from graphs to focus on more targeted and novel compounds.

To identify novel therapeutic drugs for CML BC, we screened three CML cell lines representing blast phase disease. All cell lines proliferated at similar rates during the 72 h incubation period (fold-changes in viability: EM-2, 2.90; K-562, 3.01; MOLM-1, 4.00). As the analyzed cell lines lacked a specific control group (such as healthy bone marrow–mononuclear cells as controls of patient samples), we used DSS values of >150 cell lines as a control. To determine the CML BC-specific sensitivity of tested drugs, we chose the 30 most selective drugs for closer comparison (Figure 1). Drug responses of nonspecific classical cytotoxic drugs were filtered out to a separate group, focusing the data analysis on more targeted and novel compounds (Supplementary Figure 1A).

The cell lines shared several drugs that fell into the top 30 selective drugs, although many individual differences occurred (Figure 1). All cell lines were predominately selectively sensitive to second- and third-generation BCR-ABL1 inhibitors (for example, nilotinib, dasatinib, bosutinib and ponatinib), although MOLM-1 cells were less selectively sensitive to them in comparison to EM-2 and K-562 cells. Other large drug class showing selective responses in all cell lines was VEGFR inhibitors (for example, tivozanib, axitinib, nintedanib and foretinib). Daporinad (NAMPT inhibitor), bryostatin 1 (PKC inhibitor) and danusertib (pan-Aurora and BCR-ABL1 inhibitor) were single drugs showing selectivity across all cell lines. EM-2 was highly sensitive to GSK269962 (ROCK1/2 inhibitor) unlike any other sample. Instead of BCR-ABL1 inhibitors, MOLM-1 cells were highly sensitive to BCL2 inhibitors (venetoclax and navitoclax) and glucocorticoids (for example, dexamethasone, methylprednisolone and prednisolone). No glucocorticoid sensitivity was seen in other cell lines.

There was no common drug selectivity pattern when comparing classical cytotoxic drugs between three tested CML cell lines (Supplementary Figure 1A). EM-2 cell line was most sensitive to cytotoxic drugs, whereas K562 and MOLM-1 were markedly less so. A few drugs (cytarabine, teniposide, topotecan and irinotecan) were shared in the top 20 of classical cytostatic drugs but none of them was effective in all cell lines. Methotrexate and pemetrexed were highly selective in EM-2 and K-562 cells but showed no activity in MOLM-1 cells. On the other hand clofarabine was effective in EM-2 and MOLM-1 cells but K-562 cells were resistant to it.

### Primary patient cells are sensitive to MEK and VEGFR inhibitors in addition to BCR-ABL1 inhibitors

**Figure 2:**
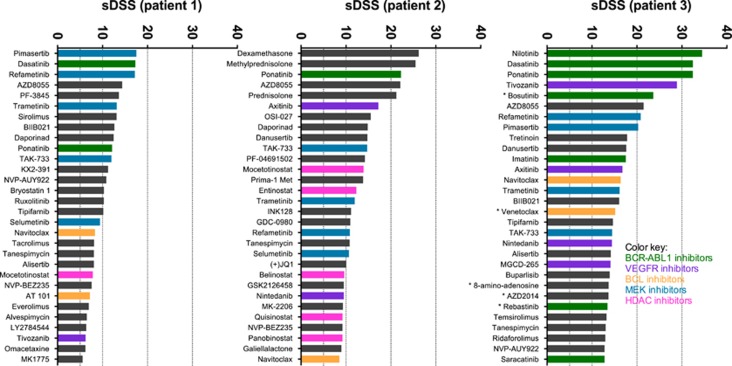
Top 30 selective DSS (sDSS) in patient samples without conventional cytostatic and cytotoxic drugs. Drugs denoted with asterisk were tested only in patient sample 3.

The top 30 comparison of drug sensitivity between primary patient cell samples was done similarly as with cell line samples. In addition to DSRT, patient samples were also sequenced, which revealed mutations in the kinase domain of the *BCR-ABL1* gene in patients 1 and 2 (E255K and T315I, respectively), whereas no BCR-ABL1 mutations were found in patient 3. Compared with the cell line data, drug responses in the primary patient cells exhibited more inter-individual variability, which could be due to the specific mutations (Figure 2). Leukemia cells of patient 1 exhibited lower levels of drug-selective responses (that is, were less drug sensitive) than other patient samples. However, the blast count in the patient sample 1 was lower than in two other patient samples (40% vs ⩾75%).

All patient samples showed a varied degree of sensitivity to BCR-ABL1 inhibitors, with ponatinib being the only BCR-ABL1 inhibitor that was effective in all patient samples (Figure 2). The cells of patient 1 that had an E255K mutation were only sensitive to dasatinib and ponatinib and showed minimal or no effect to other BCR-ABL1 inhibitors (for example, imatinib and nilotinib). Patient 2 had a T315I mutation and hence the patient cells were resistant to all primary BCR-ABL1 inhibitors except ponatinib and axitinib. Cells derived from patient 3 (with no mutation affecting BCR-ABL) were highly sensitive to all BCR-ABL1 inhibitors and the drug response profile resembled that of the TKI-sensitive cell lines (EM-2 and K-562).

A group of MEK inhibitors (refametinib, trametinib and TAK-733) were among the top 30 most selective drugs in all three primary CML BC cases. VEGFR inhibitors were also effective at inhibiting the growth of cells derived from patient samples, whereas almost no effect was observed in the cell lines. In addition to MEK and VEGFR inhibitors, AZD8055 (mTOR kinase inhibitor) and navitoclax were effective in all patient samples, AZD8055 being within six most selective drugs in all cases. Daporinad was highly selective in two patient samples and also selective in the sample of patient 3.

Leukemia cells from patient 2 displayed sensitivity to glucocorticoids. In concordance, this patient had a bi-phenotypic leukemia (both B-lymphoid and myeloid markers), whereas other patients had myeloid BC.

Between individual patients a large number of shared selective drugs (for example, vincristine, cytarabine, docetaxel and teniposide) were found among the top 20 when drug responses of classical cytotoxic drugs were compared (Supplementary Figure 1B). However, none of these exhibited high activity in all patient samples.

### Correlation of cell line and primary cell data

The DSS values of individual cell lines correlated closely with each other (EM-2 vs K-562, *r*=0.887; EM-2 vs MOLM-1, *r*=0.816; K-562 vs MOLM-1, *r*=0.775), but DSS of primary patient cells exhibited more variability (patient 1 vs patient 2, *r*=0.703; patient 1 vs patient 3, *r*=0.815; patient 2 vs patient 3, *r*=0.732). Similarly, the correlation between individual patient samples and cell lines showed high degree of variability: DSRT results from patient 3 had the best (MOLM-1, *r*=0.829; K-562, *r*=0.793; EM-2, *r*=0.842) and from patient 2 the worst correlation with the cell lines (MOLM-1, *r*=0.695; K-562, *r*=0.548; EM-2, *r*=0.597). Thus, patient samples with higher overall drug sensitivity seemed to correlate better with cell lines.

**Figure 3:**
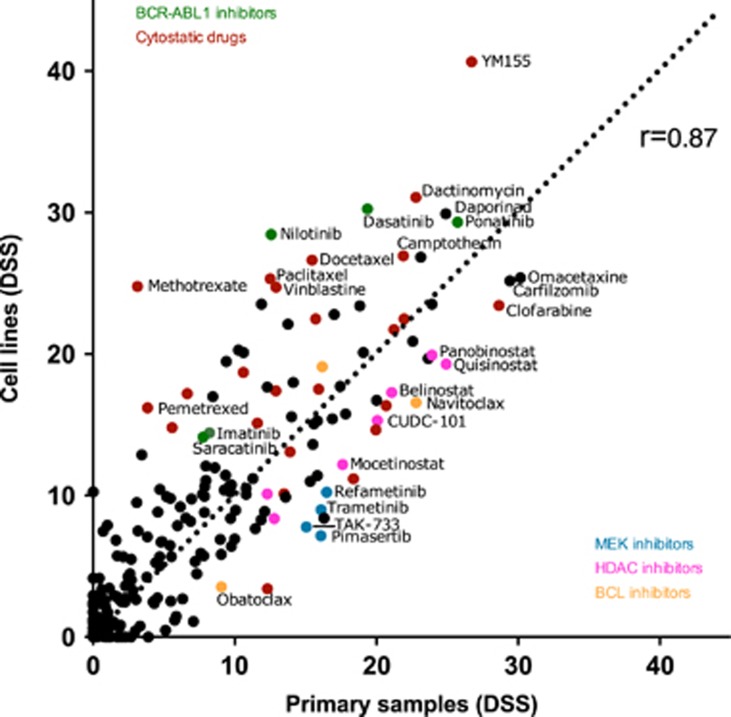
A scatter plot showing correlation (Spearman *r*=0.87, *P*>0.0001 (two-tailed)) between the average DSS values of cell lines and BC patient samples. DSS values represent the potency of drug. Only drugs that were tested on every sample, were included in the analysis (*n*=255).

Although there was individual variability, the average DSS of cell lines and primary patient cells correlated relatively well (*r*=0.87, Figure 3). The scatter plot shows that cell lines tend to be more sensitive to most drugs than primary patient cells, likely due to higher proliferation rate of cell lines in comparison with patient samples. This was especially seen with nonspecific cytostatic drugs (for example, antimitotics). BCR-ABL1 inhibitors were also more effective in cell lines, which can be explained by TKI-resistant mutations in two patient samples. In contrast, MEK, HDAC and BCL2 inhibitors were more effective in primary cell samples (Figure 3).

### Drug responses from CML samples were distinct from other leukemias

To compare drug responses on a wider scale, we performed two separate clustering analyses in which CML BC (cell line and primary) samples were compared with primary samples from different types of leukemia.

**Figure 4:**
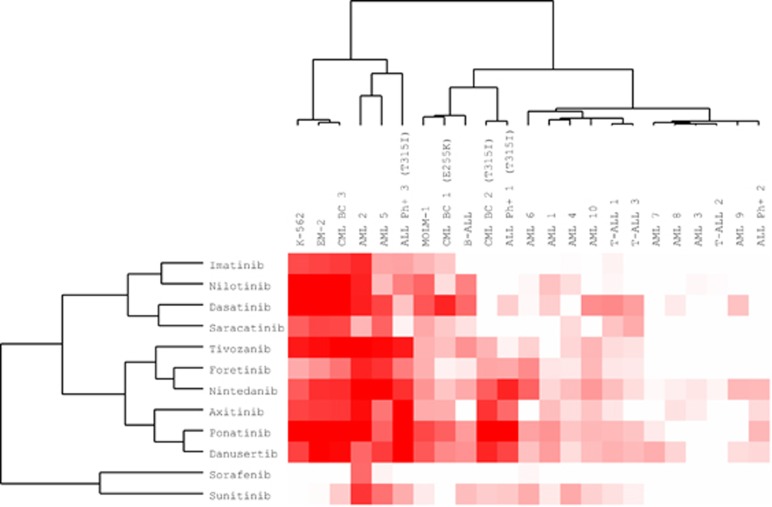
DSS of various TKIs were used in this clustering analysis to compare CML BC cell line and primary sample responses with other types of leukemia. The intensity of color indicates the drug sensitivity.

For the first cluster analysis we focused only on a small set of BCR-ABL1 and VEGFR inhibitors (Figure 4). In second cluster analysis we compared the samples with a wider set of drugs filtering out drugs that were not used in all drug screens or showed low activity in every sample (Supplementary Figure 2).

In the first analysis CML BC cell line and patient samples clustered relatively close to each other and separately from the majority of other type of leukemia samples (Figure 4). EM-2, K-562 and patient 3 clustered closely together but TKI-resistant samples (MOLM-1, patient 1 and 2) clustered further away to a different subgroup with B-ALL and T315I-mutated Ph+ ALL samples. Similarly when the wider set of drugs was used in the clustering analysis, all CML patient samples and cell lines clustered in one of the two major subclasses together with TKI-resistant Ph+ ALL samples and a few AML patients (Supplementary Figure 2).

### CGP and CCLE data correlation

The CGP study screened two cell lines (EM-2 and K-562) and 47 drugs that matched with our study. The CCLE study had only 1 matching screened cell line (EM-2) and 14 matching drugs. The drug responses of CGP EM-2 had moderate correlation (*r*=0.68, *P*<0.0001) but the drug responses of K-562 correlated poorly with our data (*r*=0.27, *P*=0.0626) (Supplementary Figures 3A and B). EM-2 drug responses of CCLE data had high correlation with our results (*r*=0.93, *P*<0.0001) (Supplementary Figure 3C).

## Discussion

In this study we assessed drug response profiles of CML BC samples by high-throughput drug testing using 295 anticancer compounds. The average drug responses of the CML BC cell lines and primary patient samples correlated well, although each sample had unique responses to specific drugs. Detailed comparison of cell line and patient sample results revealed a number of compounds (for example, VEGFR, NAMPT and MEK inhibitors) with high activity across all samples.

Recently, the Sanger and Broad institutes performed two separate large studies (CGP and CCLE, respectively) where they linked genomic profiles of various cancer cell lines to pharmacological responses.^10, 11^ In both studies the EM-2 and K-562 cell lines were included in the analysis and also common TKIs were tested. A meta-analysis of these two studies has shown some inconsistencies between the two data sets.^12^ On the basis of the comparison between our results of the EM-2 and K-562 cell lines with these two data sets, our results appeared to correlate better with the CCLE data, but the comparison was not balanced as the CGP data contained 2 matching CML BC cell lines and 47 matching drugs, whereas with CCLE data only 14 matching drugs and 1 cell line was common.

Our cell line results indicate that BCR-ABL1-positive CML cell lines are mostly sensitive to TKIs targeting ABL1. EM-2, a myeloid CML cell line with multiple copies of Philadelphia (Ph) chromosome and low tendency for differentiation, was not only highly sensitive to TKIs, but also in general was more sensitive to other drugs than other cell lines. K-562 cell line undergoes erythroid and mega-karyocytic differentiation, although it does not carry a classical Ph chromosome.^6, 8^ The high response to TKIs of K-562 cells was similar to EM-2 cells but otherwise K-562 cells were more resistant. The drug responses of MOLM-1 differed most from the other two cell lines, as TKI responses were clearly lower than in EM-2 or K-562. MOLM-1 is characterized as a mega-karyocytic cell line and in addition to the Ph chromosome with an e13-a2 *BCR-ABL1* fusion gene, it carries the inv(3)(q21q26) chromosomal aberration, which leads to overexpression of ecotropic viral integration site 1 (*EVI1*) gene.^7, 13^ High *EVI1* expression has been linked with poorer prognosis in myeloid malignancies and TKI resistance, which could be one explanation for poorer TKI sensitivity in MOLM-1 cells.^14^ Instead, MOLM-1 cells were highly sensitive to glucocorticoids, which has not been described previously.

Comparison of drug responses across all samples revealed drugs and drug classes, which deserve further investigation in a clinical setting. VEGFR inhibitors as a drug class exhibited activity in all samples, although the effect of individual VEGFR inhibitors varied from sample to sample. In our study, the most potent VEGFR inhibitors were tivozanib and axitinib, both of which are used in the treatment of renal cell carcinoma. Food and Drug Administration has approved axitinib but tivozanib is still in phase III studies. Elevated plasma levels of VEGF have been found in patients with AML and CML, and increased bone marrow vasculature has especially been noted in CML.^15^ Furthermore, lower levels of VEGF at the diagnosis have been shown to be associated with better prognosis in CML.^16^ Recently axitinib was shown to possess high affinity to T315I-mutated BCR-ABL1,^17^ which may explain the high activity in patient sample 2.

A small number of other drugs with various mechanisms of action proved to be effective in most samples. Daporinad (AKA APO866), a NAMPT inhibitor, was a top 10 hit in most samples. It is currently in phase II for various types of cancer, but not yet clinically tested in CML. Daporinad was the only NAMPT inhibitor tested in our assay but new promising compounds with the same mechanism are emerging from other screenings.^18, 19^

Danusertib (AKA PHA-739358) was the most effective aurora kinase inhibitor in our study. It has a dual role as it also inhibits BCR-ABL1 with affinity also to T315I-mutated isoform.^20^ Most of the samples were highly sensitive to danusertib, including the T315I-mutated sample of patient 2. The lack of other aurora inhibitors among most selective drugs suggests that the activity of danusertib is mostly due to BCR-ABL1 inhibition. Danusertib has been tested in clinical trials on imatinib refractory CML patients but no results have been published yet.

Interestingly, navitoclax exhibited the best activity with the patient samples compared with other BCL2 inhibitors in our assay. Similarly, venetoclax (ABT-199) was also highly effective in two samples (MOLM-1 and patient 3), but it was not tested in patient samples 1 and 2. The efficacy of BCL2 inhibitors in BC CML has recently been suggested also in other studies, which have shown that the inhibition of Bcl-2/Bcl-xL induce apoptosis of quiescent CML progenitor cells^21^ and that the combination of TKIs and BCL2 inhibitors may have synergistic effects in TKI-resistant patients.^22^

MEK inhibitors were highly selective in all patient samples but they lacked activity in the cell lines. We compared the phosphorylation of MAPK/ERK pathway of patient sample 2 (data not shown) with our recently published CML cell line phosphorylation data.^23^ MEK1/2 was more phosphorylated in cell lines but the phosphorylation of downstream targets ERK1/2 and CREB was more pronounced in patient sample. It is difficult to draw conclusions from only one patient sample, but this may implicate a MAPK/ERK pathway having a more central role in primary patient cells.

In conclusion, we characterized *ex vivo* drug responses of CML BC cell line and patient primary samples using a high-throughput drug testing method. The drug responses in TKI-resistant patient samples matched the clinical characteristics and disease course. New promising candidate compound classes such as VEGFR, NAMPT and MEK inhibitors were identified for BC CML. These drugs were highly sensitive in most samples (including TKI resistant patient samples) warranting their further evaluation as combination regimens and paving the way for proof-of-concept clinical studies.

## Figures and Tables

**Table 1:** Patient characteristics

*Patient*	*Age at diagnosis (years)*	*Karyotype at diagnosis*	*Diagnosis, disease state and phenotype at sampling*	*Therapy prior sampling*	*Time from diagnosis to sampling (months)*	*BCR-ABL1* *mutations at sampling*
Patient 1	34	t(9;22)(q34;q11)	Chronic myeloid leukaemia in second blast crisis (myeloid)	Imatinib, dasatinib, interferon, chemotherapy	20	E255K
Patient 2	35	(9;22)(q34;q11)	Chronic myeloid leukaemia in first blast crisis (bi-phenotypic)	Imatinib, dasatinib, chemotherapy	2	T315I
Patient 3	40	(9;22)(q34;q11)	Chronic myeloid leukaemia in first blast crisis (myeloid)	Imatinib, dasatinib	11	No

## Notes

SM has received honoraria and research funding from Novartis and Bristol-Myers Squibb and research funding from Pfizer. KP has received research funding and honoraria from Novartis, Bristol-Myers Squibb, Pfizer and Celgene.
