# Supplementary material for: Novel drug candidates for blast phase chronic myeloid leukemia from high-throughput drug sensitivity and resistance testing
Source: Blood Cancer J. 2015 May 1;5(5):e309–. doi: 10.1038/bcj.2015.30 (PMC4423219; doi:10.1038/bcj.2015.30)
Supplement: Supplementary Information [file bcj201530x1.pdf]

Novel drug candidates for blast phase chronic myeloid leukemia from high-throughput drug sensitivity and resistance testing

Supplementary appendix

Table of contents

Supplementary tables 2
Supplementary table 1 2
Supplementary figures 3
Supplementary Figure 1 3
Supplementary Figure 2 4
Supplementary Figure 3 5

Supplementary tables

Supplementary table 1

Full list of drugs in drug collection used in this study is provided as separate excel-file.

Supplementary figures

Supplementary Figure 1

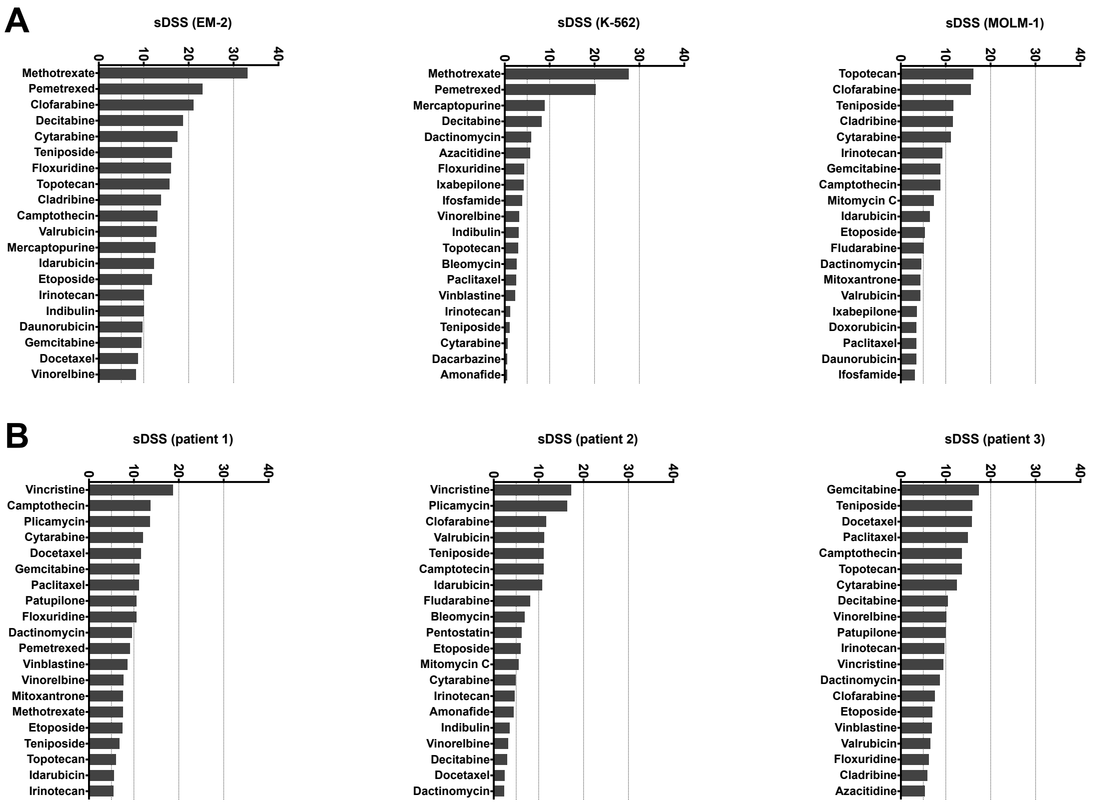

Supplementary figure 1. Top 20 cytostatic drugs in cell lines (A) and patient samples (B).

Supplementary Figure 2

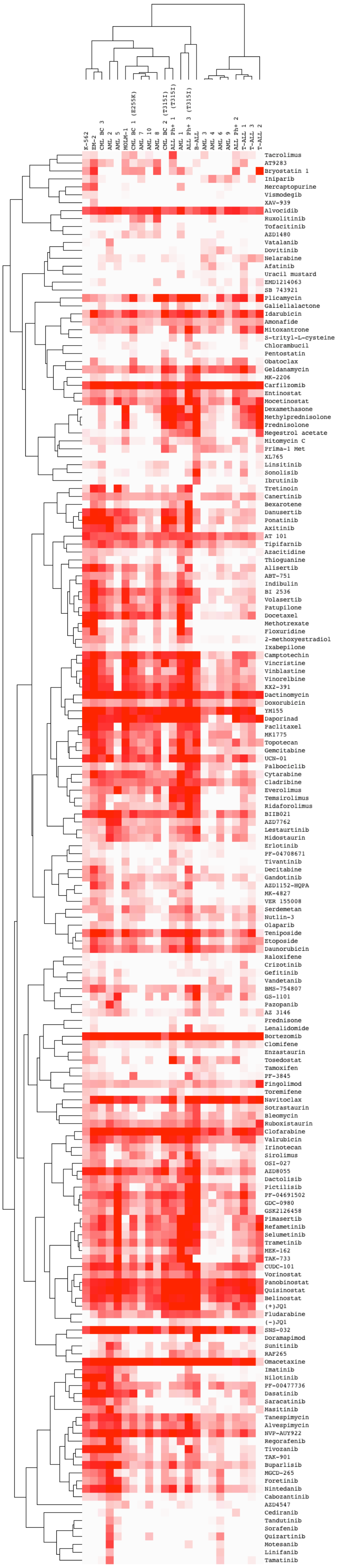

Supplementary figure 2. Clustering analysis of drug responses in cell lines and in primary samples from different leukemia. Drugs that were not used in all drug screens or showed low activity (DSS < 5) in every sample were excluded from the analysis.

Supplementary Figure 3

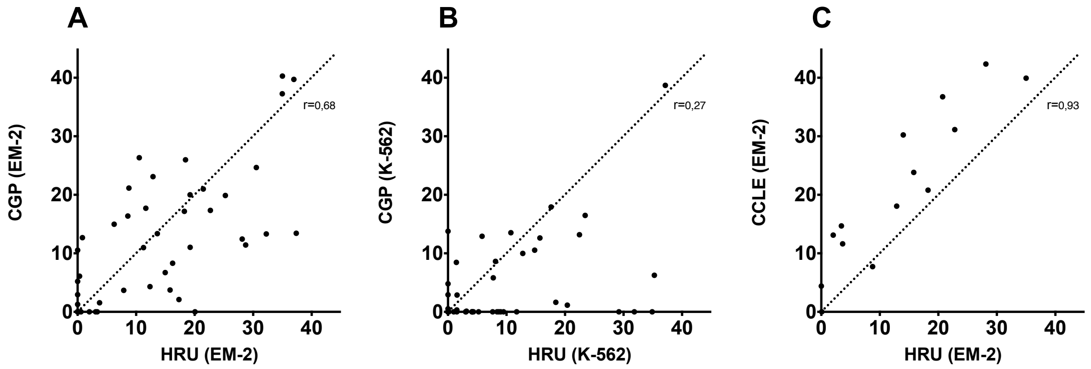

Supplementary figure 3. Scatter plot comparison of our data (HRU) with Cancer Genome Project (CGP) and Cancer Cell line Encyclopedia (CCLE) data. DSS was calculated from CGP and CCLE data and used in correlation analysis. Only EM-2 and K-562 cell lines were compared with CGP data (A and B) and EM-2 with CCLE (C).
